# DNA Polymerase Conformational Dynamics and the Role of Fidelity-Conferring Residues: Insights from Computational Simulations

**DOI:** 10.3389/fmolb.2016.00020

**Published:** 2016-05-27

**Authors:** Massimiliano Meli, Marko Sustarsic, Timothy D. Craggs, Achillefs N. Kapanidis, Giorgio Colombo

**Affiliations:** ^1^Computational Biochemistry Group, Istituto di Chimica del Riconoscimento Molecolare, National Research Council of ItalyMilano, Italy; ^2^Clarendon Laboratory, Department of Physics, Biological Physics Research Group, University of OxfordOxford, UK

**Keywords:** DNA polymerase I, molecular dynamics simulations, replication fidelity, molecular recognition, allostery

## Abstract

Herein we investigate the molecular bases of DNA polymerase I conformational dynamics that underlie the replication fidelity of the enzyme. Such fidelity is determined by conformational changes that promote the rejection of incorrect nucleotides before the chemical ligation step. We report a comprehensive atomic resolution study of wild type and mutant enzymes in different bound states and starting from different crystal structures, using extensive molecular dynamics (MD) simulations that cover a total timespan of ~5 ms. The resulting trajectories are examined via a combination of novel methods of internal dynamics and energetics analysis, aimed to reveal the principal molecular determinants for the (de)stabilization of a certain conformational state. Our results show that the presence of fidelity-decreasing mutations or the binding of incorrect nucleotides in ternary complexes tend to favor transitions from closed toward open structures, passing through an ensemble of semi-closed intermediates. The latter ensemble includes the experimentally observed ajar conformation which, consistent with previous experimental observations, emerges as a molecular checkpoint for the selection of the correct nucleotide to incorporate. We discuss the implications of our results for the understanding of the relationships between the structure, dynamics, and function of DNA polymerase I at the atomistic level.

## Introduction

DNA polymerases are molecular machines responsible for the replication and maintenance of cellular genomes (Kunkel and Bebenek, 2000). DNA replication proceeds through a mechanism whereby the parental duplex DNA is separated into its component strands, each of which provide a template for DNA polymerase. The template bases are matched to incoming deoxyribonucleotides (dNTPs) according to Watson-Crick base-pairing, and sequentially incorporated into the growing strand, leading to a new complementary duplex DNA (Joyce and Benkovic, 2004; Joyce et al., 2008). Bacterial DNA polymerase I (Pol) is one of the most widely studied polymerases, and an excellent model for understanding its function and mechanisms (Johnson et al., 2003). In *Escherichia coli*, Pol is involved in lagging-strand replication, as well as repair of damaged DNA. The protein is a multifunctional enzyme that contains three different activities, namely DNA polymerase, 3′-5′ exonuclease, and 5′-3′ endonuclease, hosted in different domains of the enzyme. The large proteolytic fragment, known as the Klenow Fragment (KF), retains both the DNA polymerase and 3′-5′ exonuclease activities, which act in concert to carry out DNA synthesis with high fidelity. The 5′-3′ endonuclease activity is involved in excision of DNA flaps that result from polymerase action during DNA repair and lagging-strand DNA replication. Moreover, Okazaki fragment processing is one of the major roles of Pol I (Patel et al., 2001).

The mechanism of polymerization, deduced from kinetic studies, entails initial formation of a binary complex of polymerase and primer-template DNA, followed by the association of a dNTP substrate to form a loose ternary Pol-DNA-dNTP complex. This complex then evolves to an activated form, which allows the chemical step to occur, involving the formation of a new phosphodiester bond and the release of a pyrophosphate(Li et al., 1998; Doublie et al., 1999; Kool, 2002; Johnson et al., 2003; Santoso et al., 2010a,b; Rothwell et al., 2013).

**Figure 1:**
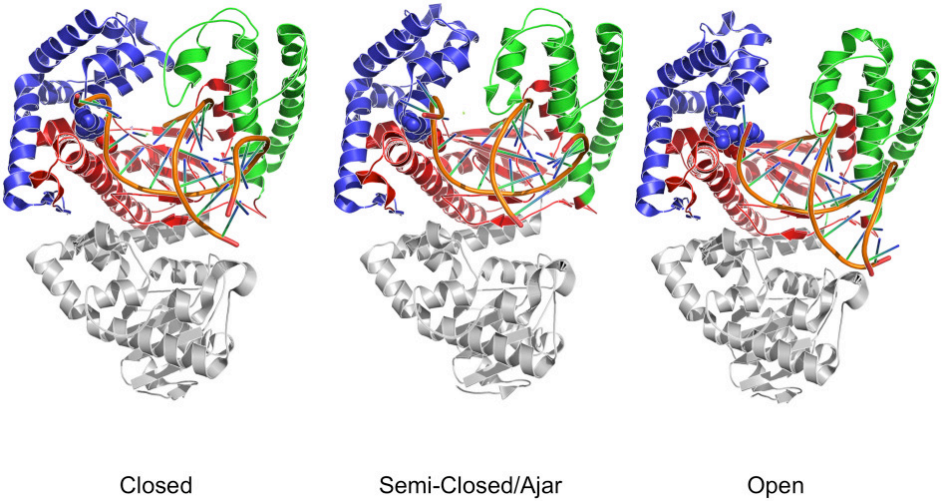
**Three dimensional crystal structures used as starting points for MD simulations**. The structures depict both DNA polymerase in three different conformational states. Different colors identify the different domains: Blue indicates the fingers; Red indicates the palm; Green indicates the thumb domain. The exonuclease domain is in light gray. Fidelity determining residues are identified as Van der Waals spheres, with E710 red and Y766 blue.

Structural studies of Pol have highlighted a common structural organization (following the description for the Klenow fragment; Ollis et al., 1985) that mimics the shape of the right hand, with palm, fingers, and thumb sub-domains (Figure 1; Li et al., 1998; Patel et al., 2001; Rothwell and Waksman, 2005; Santoso et al., 2010a). These models provide a structural basis for the observed enzymatic activity: first, binding of the duplex DNA to the polymerase causes the thumb to adapt to the DNA without major conformational changes. Second, dNTP binding to the binary complex triggers the rotation and translation of the fingers from the open to a closed conformation primed for catalysis. While the open form facilitates translocation, the closed form ensures a grip by the enzyme on the substrate. The open/closed transition in Pol is functionally significant: the closing of the fingers and thumb to form a tight binding pocket allows exclusion of incorrectly paired dNTPs, and therefore underpins the fidelity of the catalyzed DNA-replication (Dahlberg and Benkovic, 1991; Kool, 2002; Johnson et al., 2003; Joyce and Benkovic, 2004; Joyce et al., 2008; Garalde et al., 2011).

Wu and Beese, using X-ray crystallography and microsecond Molecular Dynamics studies of *Bacillus stearothermophilus* Pol, characterized the conformations preceding the chemical ligation step of the enzymatic reaction (Warren et al., 2009a,b; Wu and Beese, 2010; Miller et al., 2014, 2015a,b). These authors were able to solve the structure in three different conformations of the open-to-closed state transition, implying the presence of an “ajar” conformation that acts as a conformational checkpoint for the pre-chemistry step of the reaction. More recently, these authors have been able to report the crystal structure of an open conformation DNA polymerase I bound to DNA with a dNTP in the active site. This structure was used as a starting point to simulate the transition to the closed conformation in the presence of a Watson-Crick base pair, characterizing the main steps determining active site assembly.

We have used single-molecule fluorescence resonance energy transfer (FRET) to examine fingers-closing in *E. coli* DNA polymerase I (KF), identifying three conformations of the fingers subdomain (Santoso and Kapanidis, 2009; Santoso et al., 2010a,b; Hohlbein et al., 2013; Evans et al., 2015).

In this context, it was shown that the enzyme reactivity and fidelity of DNA replication strongly depends on the identity of the amino acids near the polymerase active site (Carroll et al., 1991; Minnick et al., 1999; Torella et al., 2011). Two particularly important and well-characterized residues are E710 and Y766 (Figure 1), both of which are universally conserved within A-family polymerases (Astatke et al., 1998a). In the binary complex, Y766 blocks the insertion site by means of a stacking interaction with the template base of the terminal base pair, whereas E710 plays a key role in positioning the incoming base by interacting with the nucleotide moiety (Astatke et al., 1998b). As the finger moves from the open to the closed conformation, Y766 rotates and is replaced by the templating base, which moves into the catalytic pocket. In this configuration, a hydrogen-bonded Y766-E710 pair forms the binding pocket, which helps to control the nascent base-pair geometry. FRET and biochemical analyses (Hohlbein et al., 2013) have shown that mutators, created by changing these residues to alanine, are characterized by a drop in fidelity, due to the perturbation of the conformational ensembles populated by the mutators compared to the wild type.

Despite these advances obtained by sophisticated experimental approaches, insight at an atomic level into the polymerase conformational changes and the impact of specific mutations of functionally oriented conformational dynamics are still elusive. To shed light on these aspects at atomic resolution, here we turn to theoretical approaches. We analyze DNA polymerase I (Pol) from *B. stearothermophilus* by extensive all-atom molecular dynamics (MD) simulations, covering a total of ~5 μs of simulation time. In particular, we focus on the Pol at different stages of the conformational mechanism preceding the chemical reaction step, investigating wild type, single, and double mutants of the protein in the open, ajar (partially-closed) and closed conformations. We also consider different ligand states, ranging from the apo enzyme to the binary and ternary complexes, with correct and incorrect dNTPs, and the effects of mutations E710A and Y766A on the properties of the conformational ensembles spanned by the protein (see Table 1 for a summary of all simulations, the labels associated to indicate each simulation and their length). Given the complexity of the conformational changes involved and the known sampling limitations of MD simulations, in this study we chose accelerated MD (aMD; Hamelberg et al., 2004, 2007; Hamelberg and McCammon, 2005) as a means to study the conformational dynamics of polymerase without the need to define a set of (most likely complex) reaction coordinates. This approach has previously been applied to study the slow dynamics of several other systems characterized by complex mechanisms, such as HIV-protease (Hamelberg and McCammon, 2005), ubiquitin (Markwick et al., 2009), the G-proteins Ras, and Rho (Grant et al., 2007, 2009, 2010a,b, 2011), and kinesin (Scarabelli and Grant, 2013).

**Table 1:** **Summary of the setup of different simulations**.

**Starting structure (PDB code)**	**Conformational state**	**Simulation length (ns)**	**Simulation type (accelerated: aMD; classical: cMD)**	**Type of complex**	**Mutant**	**Simulation label**
1L3U	Open	500	aMD	Binary (DNA)	WT	Open-aMD1
		200	cMD	Binary (DNA)	WT	Open-cMD1
1LV5	Closed	200	aMD	Ternary	WT	Closed-aMD1
		200	aMD	Ternary	E710A	E710A-aMD
		200	aMD	Ternary	E710A/Y766A	E710A/Y766A-aMD
		200	aMD	Ternary	Y766A	Y766A-aMD
		200	aMD	Ternary	Y766F	Y766F-aMD
		200	cMD	Ternary	WT	Closed-apo-CMD
		200	cMD	APO	WT	Closed-apo-CMD
		254	aMD	APO	WT	Closed-apo-aMD
		200	aMD	APO	Y766A	Y766A-apo-aMD
		200	aMD	APO	E710A	E710A-apo-aMD
		315	aMD	Binary (DNA)	WT	Closed no dNTP-1
		200	aMD	Binary (DNA)	WT	Closed no dNTP-2
		200	aMD	Binary (DNA)	E710A	E710A-Closed no dNTP-
		200	aMD	Binary (DNA)	Y766A	Y766A-Closed no dNTP
3HP6	Ajar	200	aMD	Ternary (non-complement Nucleotide)	WT	Ajar-non-complement-aMD1
		200	aMD	Ternary (non-complement nucleotide)	WT	Ajar-non-complement-aMD2
		200	cMD	Ternary (non-complement nucleotide)	WT	Ajar-non-complement-cMD1
		200	aMD	Binary (DNA)	WT	Ajar no dNTP
		279	cMD	Ternary (complement Nucleotide)	WT	Ajar-complement-cMD
		200	aMD	Ternary (complement Nucleotide)	WT	Ajar-complement-aMD

We choose here a comparative analysis approach, which exploits accurate descriptions of the different proteins' internal dynamics, to investigate and characterize the salient traits of the functional-oriented internal dynamics of the different mutators, starting from different structures. We thus integrate different structural, dynamic and energetic analyses of the trajectories (Morra and Colombo, 2008; Morra et al., 2009, 2010b; Scarabelli et al., 2010; Genoni et al., 2012; Morra et al., 2012), in order to shed light on the implications of amino acid substitutions for conformational changes, highlighting important structural and dynamic features of potential functional relevance. The simulations provide atomic-level evidence for the dynamic linkage between different regions of the protein that may underlie functional modulation and that are not directly apparent from the static X-ray structures.

## Results and discussion

### Mapping different wild-type polymerase I conformational states

We set out to map the conformational space explored by WT polymerase, starting from the open, ajar (partially-closed), and closed conformations. To this end, the structural ensemble spanned by the simulations was compared to the distinct conformations obtainable from experimental crystal structures. Principal Component Analysis (PCA) was used to assess the major conformational differences.

**Figure 2:**
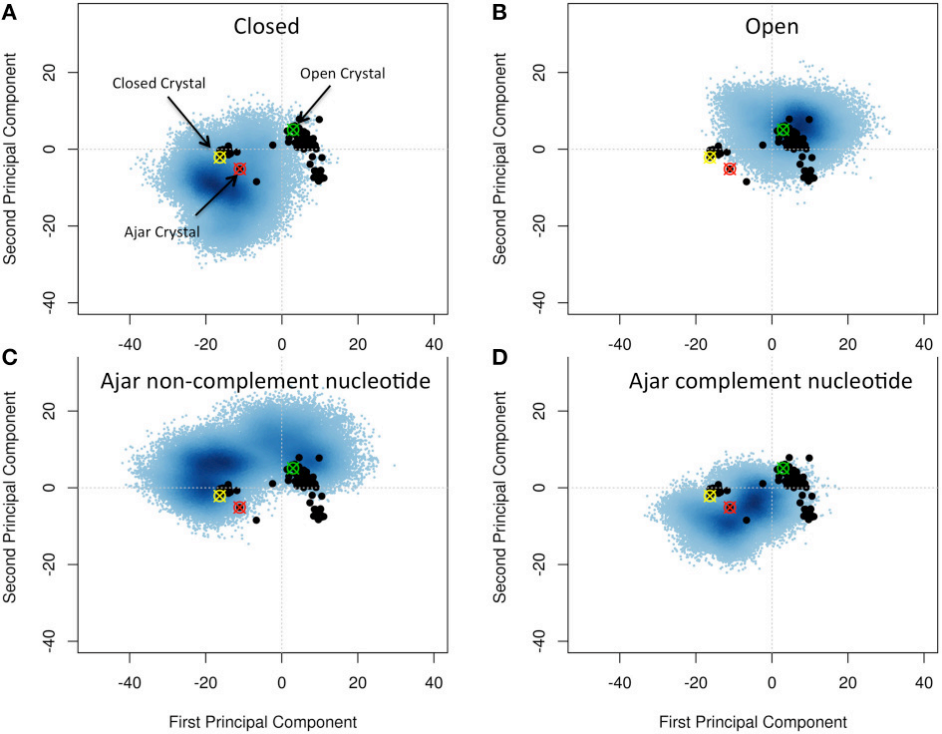
**Characterization of the essential subspaces covered by the different simulations**. The black dots indicate the experimental crystal structures, while the blue cloud recapitulates the conformational space spanned by the simulations. The starting crystal structures are indicated by colored-crossed circles: Yellow, Closed; Red, Ajar; Green, Open. **(A)** Closed simulation; **(B)** Open simulation; **(C)** Ajar with non-complementary nucleotide simulation; **(D)** Ajar with complementary nucleotide simulations.

First, we extracted 91 structures of Klenow Fragment (KF) homologs from the Protein Data Bank (see Table S1) and subjected them to interconformer PCA, to define the main directions along which principal functionally-related motions may take place, using the approach described by Scarabelli and Grant (Grant et al., 2006; Scarabelli and Grant, 2013). Over 75% of the total mean-square displacement (or variance) of atom positional fluctuations is captured in two dimensions and over 88% in three dimensions. These first Principal Components (PCs) provide an initial compact description of the main traits of conformational evolution of the system (see Figure 2). In particular, the first component, which accounts for 65% of the variance, describes a closure motion of the exo and thumb domains, which move coordinately toward each other. The fingers domain moves into the opposite direction with respect to the thumb, in a direction parallel to that of the principal axis of the bound DNA. The second component, which accounts for 10% of the variance, shows the fingers domain closing on DNA, accompanied by a torsion of the thumb domain around its main axis.

Projecting all the different experimental structures retrieved from the PDB on the plane associated with the first two PCs displayed two main basins corresponding to the closed and open structures, bridged by the ajar (partially-closed) structural basin.

Projection of the trajectories onto the two main PCs obtained from the analysis of the crystal structures showed that, starting from the closed (Figure 2A) and open (Figure 2B) states, the protein could sample a wide region of conformations around those captured in original crystal structures (Figures 2A,B). In some cases, the structures from the open state basin, in a binary complex with DNA, evolved toward the basin of closed conformations, as shown by the significant structural similarity between sub-parts of the two trajectories, based on RMSD calculations (Figure S1; Supplementary Material RMSD of open vs. closed). At the same time the trajectories from the closed structures partially evolved toward the ajar and open states. This indicated that the conformational landscapes of the open binary complex and of the closed state could partially overlap. The closed and open conformations also showed a significant overlap with the ensemble defined by the ajar trajectory.

**Figure 3:**
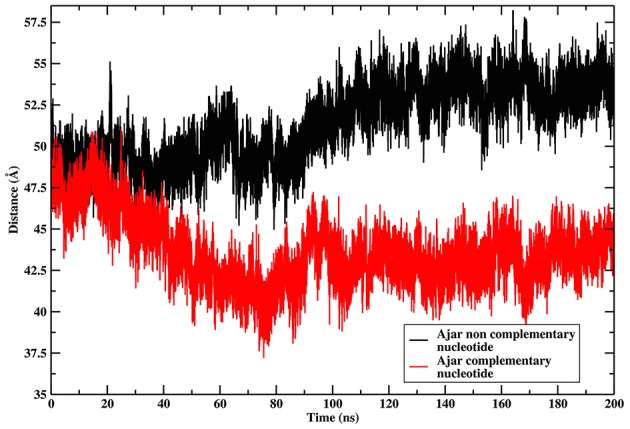
**Time evolution of the distance between the residues corresponding to the positions of FRET probes**. The graph reports on the time evolution of the distance between the residues corresponding to the positions of the FRET probes in the Ajar conformations, indicating the different conformational behavior in the presence of the correct nucleotide (red-line, closure motion), or in the presence of the incorrect nucleotide (black-line, opening motion).

The ajar system, first simulated in the presence of the non-complementary nucleotide as derived from the crystal structure (dTTP; Figure 2C), sampled a wide region of space overlapping with both the closed and open conformations. Analysis of the dynamics showed that the structure initially tended to move toward the closed state, albeit without fully populating that basin. Next, the protein moved back toward the open ensemble. Interestingly, after modeling the correct complementary nucleotide (dGTP) to be incorporated in the binding site, the simulation showed a clear transition from the ajar conformation toward the closed state (Figure 2D). Consistently, the time evolution of the distance between residues K498 (in the fingers domain) and V692 (in the thumb domain), corresponding to the residues labeled for experimental FRET analysis (respectively, K550 and L744 in KF), showed a differential behavior as a function of the bound dNTP (Figure 3): in the presence of the complementary nucleotide, the distance between the probes decreased (Figure 3, red line), indicating a transition toward a closed state. In contrast, the presence of the non-complementary dNTP pushes the protein toward a more open state, though without reaching the “fully” open conformation most likely due to sampling limitations. In this framework, the ajar state emerges as a conformational check-point for the type of nucleotide to be incorporated (Wu and Beese, 2011; Wang et al., 2012). It is interesting to note, at this point, that these results are complementary to those reported by Miller et al. (Miller et al., 2014, 2015a,b): indeed, these authors observed opening of the protein in the absence of nucleotide, using long time scale classical MD. As a further check, we thus ran simulations of the ajar and closed conformations in binary complex with only the template DNA: consistent with what observed by Miller and coworkers, the protein showed a spontaneous evolution toward open conformations (Miller et al., 2014, 2015a,b; Figure S2).

These results indicate that the main collective displacements evident in the distribution of crystal structures could be accessed during MD simulations of different structures and ligand states of the protein. The ajar conformation emerges as an intermediate checkpoint structure on the path between the open and closed conformations, being able to evolve both toward the closed and the open states. In this context, the protein features a sensing mechanism for the (combination of) bound ligand(s) that relays the signal encoded by the nucleotide into the selection of a specific conformational state.

### Effects of mutations

Previous experimental evidence (Hohlbein et al., 2013), based on FRET and single-molecule experiments, showed that specific mutations impact on the fidelity of Pol by perturbing the population distribution of open and closed states. We computationally performed the mutations (E710A; Y766A; the double mutant E710A/Y766A; and Y766F) in the closed structure of Pol in complex with DNA and the correct complementary nucleotide (dGTP), and investigated their effect on Pol conformational dynamics. Our goal here is to shed light on details of polymerase internal dynamics induced by specific mutations that may perturb the closed state structural stability and thus have an impact on the open/closed equilibrium.

**Figure 4:**
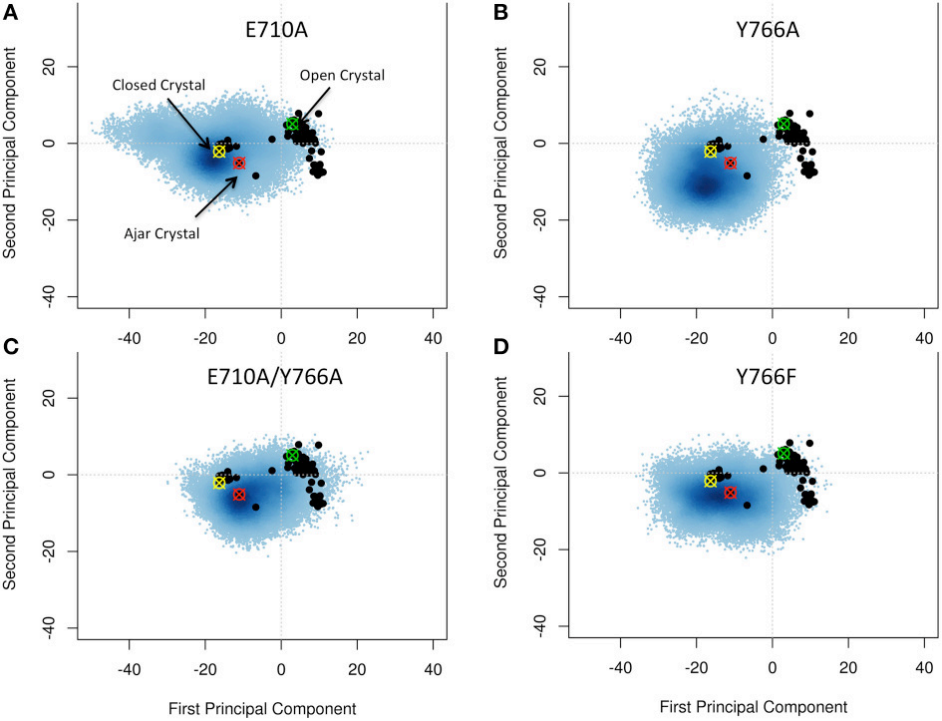
**Characterization of the essential subspaces covered by the fidelity modulating mutators simulations**. The black dots indicate the experimental crystal structures, while the blue cloud recapitulates the conformational space spanned by the simulations. The starting crystal structures are indicated by colored-crossed circles: Yellow, Closed; Red, Ajar; Green, Open. **(A)** E710A simulation; **(B)** Y766A simulation; **(C)** Double mutant simulation; **(D)** Y766F simulation.

Starting from the closed conformations, and using the same PCA-based metrics described above, single mutants E710A and Y766A, while principally populating an ensemble corresponding to the initial closed state, evolved toward the conformational basin observed for the ajar state (E710A in particular), populating structures that could reasonably be considered to connect the closed with the open states (Figures 4A,B). The double mutation E710A/Y766A (Figure 4C) appears to have the most pronounced effect on the principal motions of the protein, pushing this mutator (starting from the closed state) to sample a larger portion of the conformational subspace visited by the wild-type open conformation. Finally, Y766F displays a behavior very similar to the wild-type, remaining mostly confined to the closed state (Figure 4D). This result is consistent with experimental observations reporting a clear stabilization of the closed conformation in the Y66F mutator (Hohlbein et al., 2013).

Overall, these results confirm a key role for the E710A and Y766A mutations in modulating the stability of the closed state of Pol, consistent with experimental FRET measurements (Hohlbein et al., 2013).

### Characterization of the internal dynamics of the protein

Next, we extended our analysis to the characterization of the internal dynamics of the protein and its responses to mutations and nucleotide variation. To this end, we made use of a recently introduced method for the characterization of dynamically coordinated groups of amino acids from MD simulations (Colombo et al., 2008; Morra et al., 2009, 2010b, 2012; Torella et al., 2010; Meli et al., 2011). In this framework, the coordination propensity (CP) between any two residues in a protein is defined as a function of their distance fluctuation. Analysis of long-range coordination propensities (CP) patterns of residue-pairs is used to pinpoint protein substructures (groups of amino acids), often belonging to distal regions of the protein, that can dynamically respond to amino acid substitutions or ligand binding. This analysis aims to define coherent patterns of residue-pair coordination, corresponding to mechanically rigid substructures, which characterize protein subdomains. The identification of the protein moduli of high internal coordination, or dynamical domains, and the characterization of the variation in their boundaries as a function of fidelity-determining mutations or of the identity of the nucleotide, can provide valuable information on functionally important aspects of protein internal dynamics (Potestio et al., 2009; Zen et al., 2009; Morra et al., 2012).

**Figure 5:**
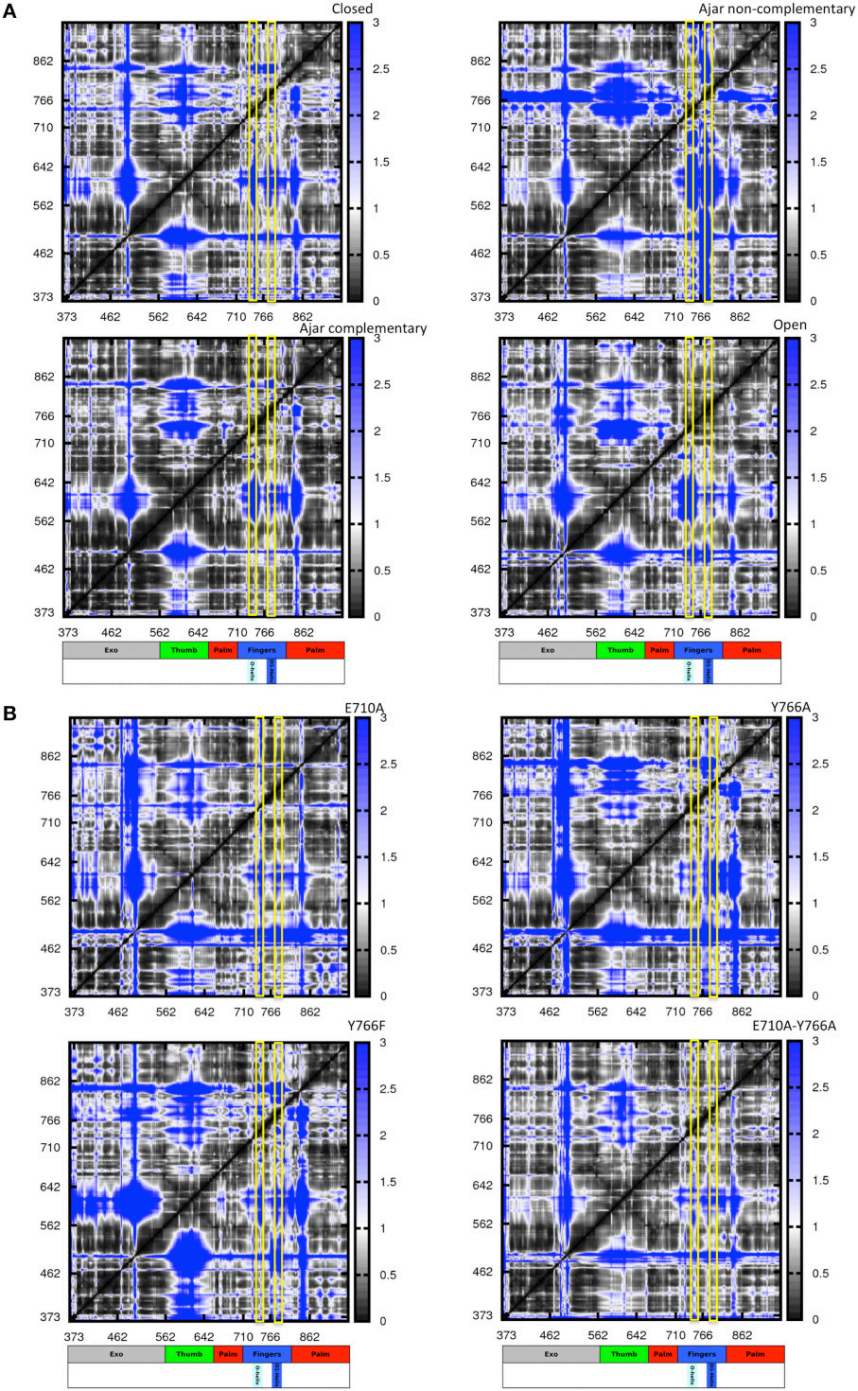
**Distance fluctuation matrices for the different structures and different sequences**. The magnitude of pairwise distance fluctuations is color coded from black (small fluctuations) to blue (large fluctuations). The different scale of pairwise fluctuations among the different molecules reflects the different degrees of internal mobility. The color coded bar represents the different domains of the protein, with helices O and O1 highlighted. **(A)** Wild type simulations; **(B)** Mutant simulations.

We first considered several ternary complexes; one consisting of polymerase in the closed state in complex with primer-template DNA and the correct complementary nucleotide, and others with polymerase in the ajar state in complex with DNA and either the complementary or non-complementary nucleotide (Figures 5A,B).

The resulting matrices yield a general qualitative picture of the internal dynamics of the protein in different states. Overall, all matrices exhibit a similar subdivision in blocks, reflecting the presence/alternation of regions of small and large fluctuations typical of multidomain proteins. Coherent patterns of low inter-residue fluctuations, which expectedly correspond to the structurally defined protein subdomains, can be observed for the regions corresponding to the 3′–5′ exonuclease, thumb, and palm domains (Figure 5A) in the trajectories of the WT closed protein.

The most distinctive feature of the internal dynamics among the ternary complexes entails the fingers domain. In the closed state, the fingers, and in particular the region spanning helices O and O1, show patterns of coordination (darker areas) with all other protein domains. Interestingly, in the ajar state containing the non-complementary nucleotide, the region containing the sensing residue Y766, helix O1 and the fingers helix immediately following it appear to be much more globally flexible and less coordinated with the rest of the protein, while the region containing E710 appears highly coordinated with the palm and thumb domains. Strikingly, substitution of the non-complementary nucleotide with the correct one leads to a dramatic change in the patterns of coordination, with the fingers sub-domain now showing strong mechanical couplings with the rest of the protein, similar to the situation initially observed for the closed state (Figure 5A).

In the open state, containing only DNA in a binary complex, the fingers are highly coordinated with the palm, defining an extended substructure combining the two regions in a coherent dynamic domain.

The peculiar dynamic behavior observed for the fingers sub-domain, and specifically for the substructure containing the fidelity-defining residues, once more corroborates the role of DNA-contacting sequence stretches in sensing the presence and identity of the ligand and transmitting this information, through mechanical coordination, to different functional protein substructures.

In this context, the analysis of mutators (ternary complexes starting from closed states with correct complementary nucleotide) shows different internal coordination patterns. In the cases of both E710A and Y766A single mutants, large areas of low coordination are evident in the matrices, indicative of overall increased conformational flexibility within the protein, even in the absence of major conformational changes for the protein. As expected, Y766F returns a coordination matrix sharing all the main traits of the WT closed state (Figure 5B).

In the case of the double mutant E710A/Y766A, the coordination network tends to resemble the one of the open state, with the fingers and palm forming a single, extended block of coordination (darker region close to the diagonal). This is consistent with the increase of spontaneously populated open-like conformations for the double mutant, previously characterized with PCA analysis, whereby the fingers/palm part can move as a coherent mechanical unit facilitating the population of the open state.

### Energetics of protein conformations and impact of fidelity impacting mutators

**Figure 6:**
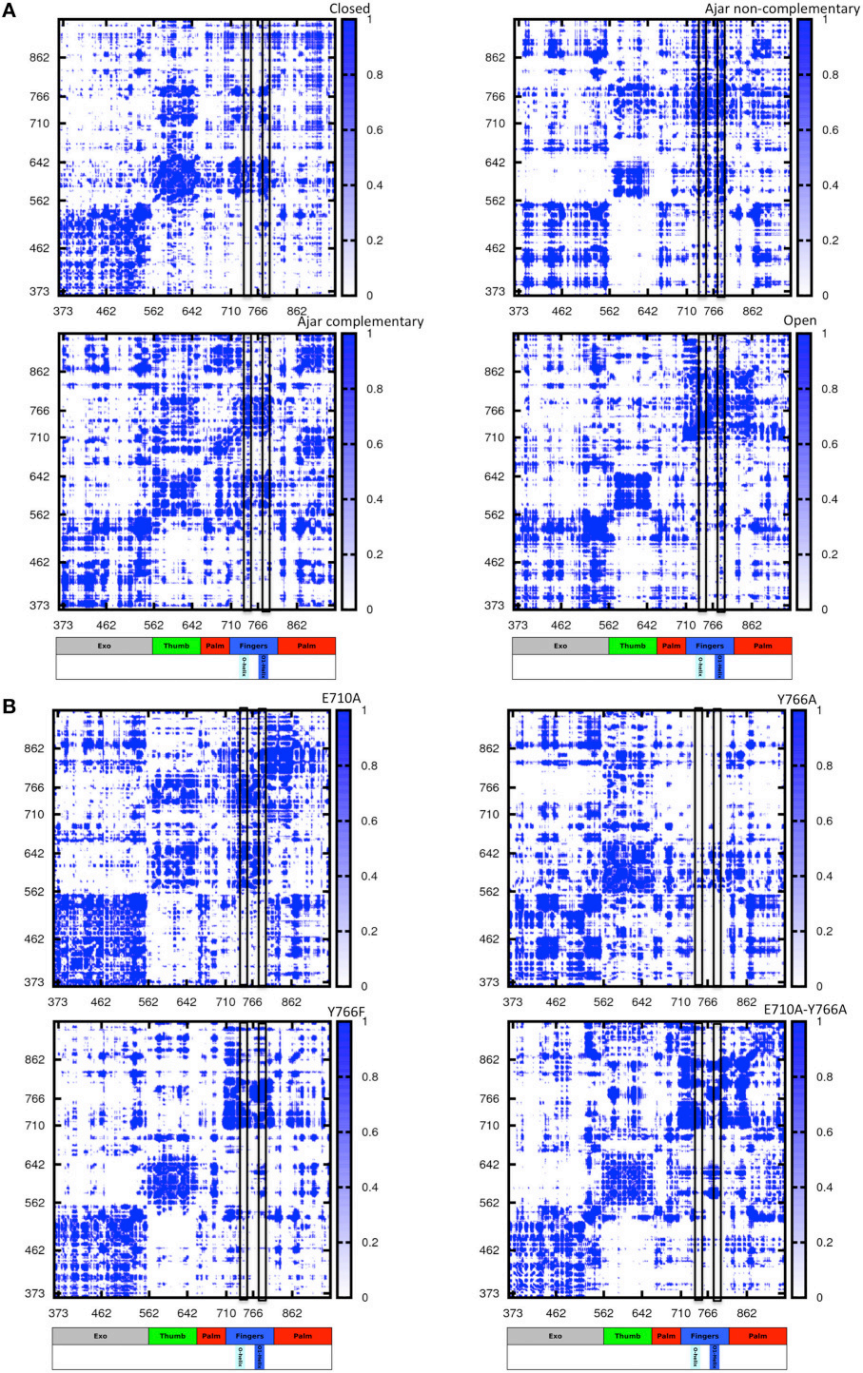
**Pair-interaction energy matrices for the different structures and different sequences**. The intensity of pairwise stabilizing fluctuations is color coded from white (no interaction) to blue (strong interactions). The different scale of pairwise interactions among the different molecules reflects the different internal organization of the proteins. The color coded bar represents the different domains of the protein, with helices O and O1 highlighted. **(A)** Wild type simulations; **(B)** Mutant simulations.

**Figure 7:**
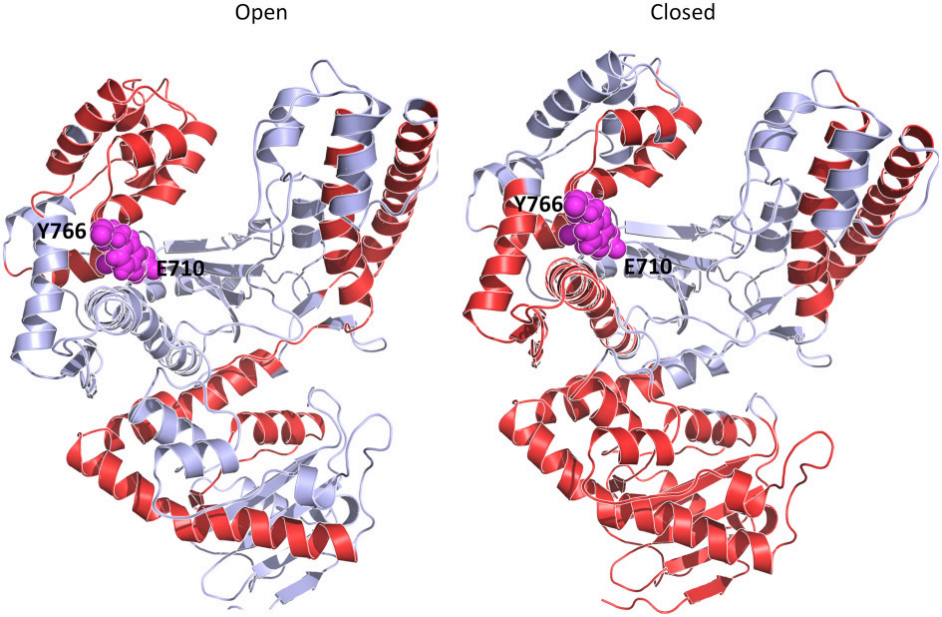
**Principal stabilizing hot spot comparison**. The stabilizing centers are highlighted on the ribbon diagram of the Polymerase I structure with the red coloring. E710 and Y766 are highlighted as magenta Van der Waals spheres.

To gain further insight into the determinants of (de)stabilization of a certain conformation in response to varying conditions, we analyzed the energetics of different trajectories using the Energy Decomposition method (Tiana et al., 2004; Morra and Colombo, 2008; Genoni et al., 2010, 2012; Scarabelli et al., 2010; Morra et al., 2014; EDM). In this approach, a simplified map of the interactions important for the stability of a certain structural ensemble can be proficiently reconstructed from the analysis of the *N*×*N* matrix (*M*) of average non-bonded interactions between pairs of residues calculated along an MD trajectory, with *N* being the number of residues (Tiana et al., 2004; Morra and Colombo, 2008; Genoni et al., 2010, 2012; Scarabelli et al., 2010; Morra et al., 2014). The main residues responsible for structural stabilization can be revealed through eigenvalue decomposition analysis of the resulting matrix. The regions determining the coupling interactions most relevant for the stabilization of the open vs. closed states, and the consequences of mutations and of binding of different nucleotides, are reported in Figure 6 and projected on the 3D structures of Pol in Figure 7.

As expected, the analysis of Pol in the closed and open conformations highlights a common subdivision in blocks, which correspond, as in the case of the coordination propensity analysis, to the classical structural subdomains (Figure 6A panels open and closed). The principal residue-residue interactions in the open conformation appear to concentrate in a limited number of sub-blocks corresponding to the two long helices of the 3′-5′-exonuclease domain, and to the principal helices of the thumb and the fingers sub-domains, in particular the region comprising E710 and Y766. The cross-domain interactions define a diffuse network extending across the whole structure. In the case of the closed conformation, the energy block comprising the two long helices of the 3′-5′ exonuclease domain extends over the whole domain. The block defined by the principal helices of the palm appeared to be strongly coupled with the two O and O1 helices of the fingers sub-domain. The block-like character of the latter, in turn, is significantly reduced compared to the open state. Interestingly, substructures of strong interactions are mostly centered on regions containing the fidelity defining residues E710, Y766.

To shed light on the role of the partially-closed structure as a checkpoint for nucleotide incorporation, we extended our energetic analysis to the ajar trajectories. The presence of the incorrect nucleotide in the active site dramatically disrupts the interaction networks between the 3′-5′-exonuclease, the palm and the thumb domains. The disruption of this interaction network, relevant for the stabilization of the closed state, can facilitate fingers opening and the consequent rejection of the incorrect dNTP.

Strikingly, the pattern of the interaction matrix observed for the closed conformation is recovered upon exchanging the incorrect nucleotide for the correct one. This result, based on energetic considerations, is consistent with the observed tendency of the ajar conformation to evolve toward the closed state in the presence of the complementary nucleotide.

Next, we investigated the effects of mutations in fidelity determining residues: in all cases changes in the interaction matrix, with respect to the WT closed situation, were evident. For E710A, the mutation results in the disruption of interaction networks around the mutated residue. In addition, the energetic blocks defining the thumb and fingers domain are less connected to the 3′-5′-exonuclease domain, and the block-like character of the fingers domain is significantly reduced.

Y766A mutation results in a dramatic disruption of the interaction networks defining the fingers domain, suggesting that the mutation causes the domain to be less internally stable than the wild-type counterpart. This could manifest in less ordered motions (lower dynamic coordination with the rest of the protein) for the fingers, consistent with the coordination propensity analysis, which could lead to a substantially decreased fidelity in the nucleotide recognition process. Interestingly, the interaction networks typical of the WT closed state are mostly recovered with the Y766F mutation, albeit with a decreased coupling between the thumb and fingers.

Finally, the interaction matrix for the double mutant E710A/Y766A returned a set of interaction patterns similar to those observed for the open state analyzed in a binary complex with DNA. It is worth noting here that the starting conformation is the closed one and that these results show the capability of the energetic analysis to capture the destabilizing effects on the closed state of mutations that favor the open one.

Interestingly, these observations are in line with results from the Thirumalai group (Zheng et al., 2005, 2006), who used statistical mechanical analysis and elastic models of polymerases, and identified the fingers (as well as part of the palm) domains as the regions that localize the networks of residues responsive to perturbations.

Next, we calculated the stability of different protein conformations and mutants, using the Molecular Mechanics-Poisson Boltzmann Solvent Accessible (MM-PBSA) approach (Lee et al., 2000). The calculations were carried out on the representative structures of the most populated structural cluster obtained from the full-length trajectories. It is readily seen that (Figure S3) the presence of fidelity-compromising mutations in the closed state induces destabilization. In contrast, mutation Y766F provides the largest stabilization of closed conformations, once more consistent with experimental observations (Hohlbein et al., 2013).

Overall, the results of energetic analyses suggest that the perturbation of the fidelity-modulating residues results in notable changes in the interaction energy patterns. This indicates that amino acid substitutions at these relevant protein hot spots translate into a modulation of the highly interconnected residue-networks defining the stability of the different states of the protein.

### MD derived picture for polymerase fidelity mechanisms

In this work, we have characterized the structural and dynamic features of Pol starting from different conformations available from X-ray crystallography, through the analysis of MD simulations. Investigating the principal traits of polymerase dynamics can be challenging due to the sampling limitations of classical all-atom MD simulations, especially in the case of complex conformational rearrangements. To overcome these hurdles and gain insight into the determinants of functional dynamics at the atomistic level, we have used accelerated MD as a suitable means to extend conformational sampling without the need of using a (combination of) pre-defined reaction coordinate(s). We subsequently carried out comparative analyses of the resulting conformational ensembles by novel methods aimed to highlight the principal traits of internal coordinated dynamics and the underlying interaction networks. These methods have been previously applied to the investigation of protein stability and dynamic changes in response to ligand binding and sequence variation (Tiana et al., 2004; Colombo et al., 2008; Morra and Colombo, 2008; Morra et al., 2009, 2010b, 2012, 2014; Genoni et al., 2010, 2012; Scarabelli et al., 2010; Torella et al., 2010; Meli et al., 2011). Pol, its complex dynamics and their response to mutations represent a relevant case for testing the reach of such methods.

The analysis of energetics and internal dynamics indicates that the closed conformation of the wild type sequence bound to the correct complementary dNTPs is the most stable, compared both to complexes of fidelity-compromised mutants with the correct nucleotide and to complexes of the wild type protein with non-complementary nucleotides. This is in line with a range of experimental data including X-ray structural analysis, biochemical and FRET-based analyses (Hohlbein et al., 2013).

Principal Component Analysis of the motions of protein mutants known to affect the fidelity of DNA replication shows a destabilization of the closed state and an evolution toward the ajar ensemble.

Starting from the ajar structure, the protein spans an ensemble of conformations that bridge the closed and open states with high sensitivity to the specific dNTP present at the active site (Figures 2, 4). Specifically, in the case of the non-complementary nucleotide, the conformations initially move toward the closed ensemble, without fully populating it, and then revert toward the direction of the open one. In contrast, the presence of the correct complementary nucleotide favors the transition toward the closed conformations; the protein can thus dynamically populate an ensemble of structures that are intermediate between fully closed and fully open structures. In this view, the results of both the simulations of fidelity-decreasing mutations and of those starting from ajar structures are consistent with the observation of intermediate-FRET states, as the pre-dominant species in solution for ternary complexes with incorrect nucleotides and in ternary complexes of low-fidelity derivatives for both correct and incorrect nucleotides (Hohlbein et al., 2013).

Miller et al. (2014, 2015a,b) studied the binary complex between Pol and DNA, starting from different conformations of the protein using long timescale MD simulations at high temperature. They showed that the binary complex tends to transition to the fully open conformation, independent of the starting conformation, revealing an intermediate state on pathway between the open and closed states. Interestingly, their simulations of Pol fingers-opening suggest the existence of an intermediate state that is distinct from the ajar state observed in the crystal structure with the non-complementary nucleotide. These results, and subsequent characterization of the open to closed conformational in a Pol ternary complex (Miller et al., 2014, 2015a,b), indicate the presence of different intermediate conformational ensembles and steps in the closing mechanism, consistent with what we have observed here. In this context, we note that our result may aptly complement the mechanistic details emerging from the work of Miller et al. who used plain long time scale MD simulations.

**Figure 8:**
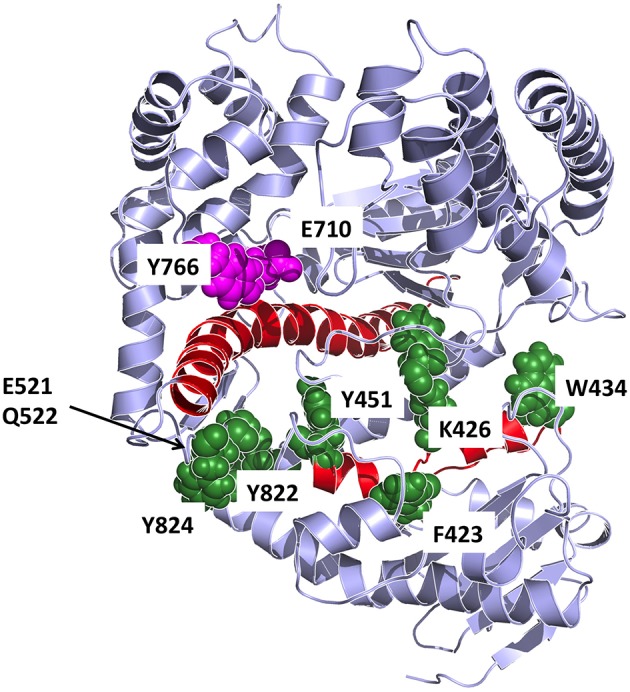
**Detailed representation of the principal interaction networks**. The residues that are most involved in conformational regulation on the basis of energetic and coordination analysis are depicted in the structure as van der Waals spheres, colored in dark green. The two helices responsible for transmitting the signal from the catalytic site are colored in red. Catalytic residues are colored in magenta.

In our work, to obtain an atomistic description of the mechanisms of stabilization of different conformations we set out to combine energetic and dynamic analyses. First, on the basis of the energy matrices we created a vector whose components are the sums of the elements in the respective matrix column (for the open and closed conformations). The components of such vector, whose length equals the sequence length, indicates the contribution of each residue in conformational stabilization (Morra et al., 2014). The use of the energy decomposition approach identifies several different regions as possible hotspots related to the stability of the open or closed conformations (some are in common; see Figure 8). The projection of the components with a value more negative than the average value of all components (more stabilizing) on the structures highlights which residues can be mutated that may have a strong impact on the structural properties of Pol (Torella et al., 2010).

Next, to pin down a limited number of residues apt to interfere with the protein's conformational dynamics, we analyzed the differential local flexibilities [*p(i)*] (see Methods) of the closed and open conformations. *p(i)* reports on the local deformation of the structures in the different conformational ensembles (Figure S4 reports the differences in the eigenvector and local flexibility components for the open and closed conformations).

The combination of these data highlighted residues that are primarily important for conformational regulation, by determining the stability of a certain structure (energy hotspots) and which are in contact with a region that shows significant differences in flexibility between the different dynamic states of the protein. Such residues are principally: F423, K426, W434, Y451, E521, Q522, F658, R822, Y824. The first three residues of the group are in the upper part of the exo domain and form a network of contacts that, in our model, can connect the exo with the thumb domain. Interestingly, F423 is part of the helix that hosts Y451. The latter is part of a tightly packed ensemble of aminoacids that contact the long helix (red in Figure 8) in the palm domain, which in turn interacts with fidelity determining E710 and Y766. The molecular model emerging from this analysis indicates a network of residues that connect the nucleotide sensing machinery (E710 and Y766) to the thumb. In this framework, the long rigid helix in the palm and the helix hosting F423 and Y451 appear to act as a mechanical transmitter of the conformational signal generated at the active site upon nucleotide binding, to distal parts of the protein. Modifications of E710 and Y766 would clearly perturb this interaction pattern and potentially result in a modification of the resulting global dynamics of the protein.

We suggest that the consideration of multiple conformational ensembles and steps on passing from the open to the closed, reactive conformation of the enzyme can generate a model reconciling the observed Pol dynamics and the roles of the ajar conformation present in X-ray structures with incorrect nucleotide. The protein in solution can fluctuate at equilibrium among different dynamic states, and binding-unbinding of a specific nucleotide may shift the conformational distribution either toward the closed state (complementary nucleotide) or away from the closed state toward the open one (non-complementary nucleotide) favoring the population of an ensemble of intermediate structures (Miller et al., 2014, 2015a,b). In our analyses, these intermediate structures also span the conformational ensemble around the ajar conformation (Figures 2, 4) and binding of a nucleotide can select and stabilize the latter state, thus bringing the protein into the structure observed experimentally by X-ray crystallography. In this framework, the ajar structure can aptly be considered as a representative of a specific subset of conformations that can be captured in crystals and that act as control checkpoints to direct the evolution of the protein dynamics toward the open or closed states.

**Figure 9:**
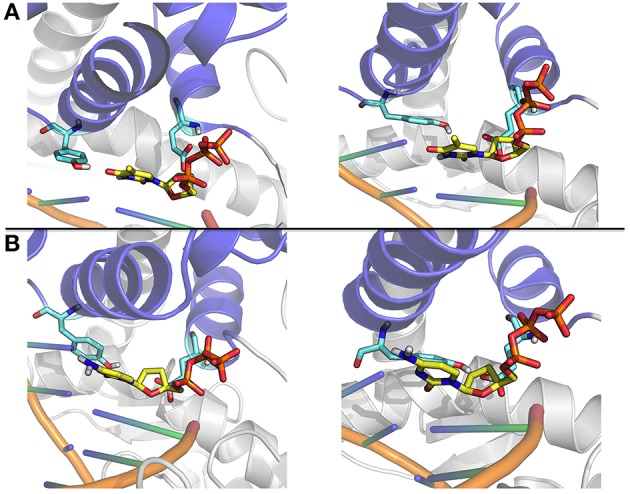
**Close up on representative structures of the active site. (A)** The representative structures of the two most populated clusters for the Ajar simulation with the non-complementary nucleotide. **(B)** The representative structures of the two most populated clusters for the Ajar simulation with the complementary nucleotide.

The simulations of mutators in the closed state, moreover, shed light on the specific roles of positions 710 and 766 in the modulation of fingers-closing transition and nucleotide selection (Figure 9). E710 regulates the local interaction patterns at the basis of the fingers-dynamics and its perturbation results in a general decrease in dynamic coordination with the rest of the protein. Y766 lies at the crossroad of an important network of interactions, connecting the protein to the incoming nucleotide and the templating DNA. The presence of an aromatic side chain at position 766 ensures the tight hydrophobic packing with the nucleotide necessary to correctly position the nucleic acid in the enzyme active site. Modification of Y to F, indeed, does not perturb either the fidelity or the recognition properties of polymerase. In sharp contrast, A at position 766 shuts down the compact network of amino acids that likely ensure the stabilization of the fingers in the closed state (Figure 9). This is particularly evident from the analysis of the simplified energy matrix derived from the energy decomposition analysis approach: such a disruption of the energetic networks relevant for the stability of the closed structure will impact on the overall conformational equilibrium of the protein, shifting it toward the semi-closed and then the open states (Figure 6).

The nucleotide sensing/regulatory role of Y766 is aptly demonstrated by the analysis of the ajar simulations. In the presence of the non-complementary nucleotide, Y766 appears to populate different possible conformations, due to non-optimal packing (Figure 9). Optimal packing is in contrast possible in the presence of the complementary dNTP, favoring the formation of a tight active site complex, necessary to allow the chemical reaction step of DNA elongation.

In summary, internal dynamics and energy decomposition analyses indicate that sequence properties and binding are coupled to conformational transitions associated with the biological functions.

## Conclusions

The regulation of internal dynamics of DNA polymerase I by specific residues or ligand binding was studied here by extensive atomistic MD simulations, covering a total time span of 5 ms. The data were analyzed within novel schemes that are aptly used to filter out the principal determinants of polymerase internal dynamics. The combined internal dynamics and energetic decomposition approaches were shown to capture the salient features of nucleotide recognition and fidelity, and their responses to perturbations such as mutations. Our analyses reveal the presence of specific patterns of internal interactions and modulation of protein dynamics. Importantly, in this context, the semi-closed ensemble of conformations was detected as a possible conformational checkpoint for nucleotide selection.

The straight-forward approach we used here may represent a novel and effective means of gaining insight into finely tuned mechanisms of functional regulation in enzymes, with implications at the practical and fundamental levels. From the practical point of view, the results of our simulations and the approaches tested herein can be used to design novel mutants with specific dynamic and recognition properties, as well as to design regulatory chemical probes that interact with specific regions crucial for the functional dynamics of the protein. From the fundamental point of view, the comparison between different sequences structures and complex states can help in understanding the molecular basis of DNA replication as well as furthering our understanding of the relationship between protein sequence, structure, dynamics, and activity in proteins.

## Materials and methods

All MD simulations and standard structural analyses were performed with the AMBER 12 suite of programs (Case et al., 2012), using the *ff12SB* force field and the TIP3P water model and the CUDA implementation for GPUs. The structural representatives of the three different conformational states of Pol were extracted from the Protein Data Bank with the following codes: 1L3U, 1LV5, 3HP6.

These structures were considered as the starting points for all the simulations reported in Table 1. Binary complexes were obtained by simply removing the coordinates of the dNTP present in the X-ray structure in the cases of 1L3U and 1LV5. In the case of the ajar structure, solved in the presence of the non-complementary nucleotide, the complex with the correct nucleotide was obtained by modeling dGTP in the binding site. All Mg^2+^ ions and water molecules present in the crystal structures were retained for the simulations. All the structures were solvated in a cubic box large enough contain the complexes and 1.2 nm of solvent on all sides.

Each system was initially minimized with a 2-step procedure: first a restrained minimization of 4000 steps was carried out, keeping the backbone of the protein, DNA and Mg ions fixed with a restraining force of 100 kcal/mol. Next an unrestrained minimization was carried out, consisting of 2000 steps of steepest descent followed by 2000 steps of conjugate gradient minimization.

Different independent trajectories were generated for each system reported in the table with different random seeds. All simulations were carried out in the NPT ensemble at 300 K and 1 atm using the Berendsen coupling algorithms (Berendsen et al., 1984), with full particle-mesh Ewald electrostatics (Darden et al., 1993). The SHAKE algorithm (van Gunsteren and Berendsen, 1977) was used to constrain all covalent bonds involving hydrogen atoms. A 2fs time step and a 10 Å cutoff were used for the truncation of VDW non-bonded interactions.

A short equilibration simulation at 300 K of 2,500,000 steps was carried out. The evaluation of the mean total energy for this short run was used to define the boost parameters for accelerated MD as described before by the McCammon (Hamelberg et al., 2004, 2007; Hamelberg and McCammon, 2005) and Grant groups (Grant et al., 2010a; Scarabelli and Grant, 2013). Indeed, in order to enhance sampling a boosting accelerated molecular dynamics (aMD) approach was employed, based on a total energy boost potential. The energy level, E, below which the boost is applied and tuning parameter, α, that modulates the depth and local roughness of basins in the modified potential, were based on previous works and on tuning of the energies based on preliminary 20 ns MD runs, and a value of 0.20 Kcal/mol was chosen in this case (see refs and AMBER manual). The E values (Kcal/mol) are: Open 15882; Closed 14883; Ajar 15306; E710A 14882; Y766A 14889; E710A/Y766A 14880; Y766F 14883.

Herein, accelerated MD (aMD) was used as a tool to speed up conformational search, using the same acceleration parameters for all simulations. Each of the analyzed structures was minimized to the nearest local minimum. This method was used to probe the conformational energy surface of the protein and compare the results to available experimental data.

We also performed reweighting of the trajectories as described by Miao et al. in Miao et al. (2014). We report the projections of the reweighted trajectories in the new version of the Supplementary Material (Figure S5).

All the structural and principal components types of analyses were carried out using the AMBERtools suite of programs.

### Distance fluctuations matrix

On each trajectory, we computed the matrix of distance fluctuations *A*:

(1) $$A_{ij}=\langle {\left(d_{ij}-\langle d_{ij}\rangle \right)}^{2}\rangle$$

where *dij* is the (time-dependent) distance of the Cα atoms of amino acids *i* and *j* and the brackets indicate the time-average over the trajectory. The *A* matrix, and various quantities derived from it, can be used to characterize the salient elasticity and plasticity properties of a protein undergoing structural fluctuations (Morra et al., 2012).

Local flexibility was calculated making use of the *A*_*ij*_ values from the coordination matrices; *d*_*ij*_ is the distance between residue i and j, the brackets indicate an avg value along the trajectory. *d*_*ij*_ is calculated only for residues along the sequence, with j ranging between i−4 and i+4.

(2) $$p\left(i\right)=\sum_{j}A_{ij}f\left(\langle d_{ij}\rangle \right)$$

### Principal component analysis

Principal component analysis (PCA) was used to analyze the relationships between superposed structures and molecular dynamics trajectory output. PCA, or Essential Dynamics (ED) has been proven to provide relevant details regarding the nature and directions of the main conformational changes in different protein families (Garcia, 1992; Amadei et al., 1993, 1996; van Aalten et al., 1997; Scarabelli and Grant, 2013). Herein, we have applied PCA to the distributions of available experimental structures of Pol and projected the structures obtained from the MD trajectories on the plane defined by the first two principal components.

PCA is based on the diagonalization of the covariance matrix, *C*, with elements *C*_*ij*_, built from the Cartesian coordinates, *r*, of the superposed structures:

(3) $$C_{ij}\;=\;<(r_{i}\;-\;<r_{i}>)(r_{j}\;-\;<r_{j}>)>$$

where *i* and *j* represent all possible pairs of *3N* Cartesian coordinates, where *N* is the number of atoms being considered. This matrix can be used to highlight protein regions that move coherently. The eigenvectors of the covariance matrix correspond to a linear basis set of the distribution of structures, called the principal components (PCs). The respective eigenvalues associated to each eigenvector define the variance of the distribution along the corresponding eigenvectors. This method describes global protein motions that are represented by the matrix eigenvectors and eigenvalues and emphasizes the amplitude and direction of dominant protein motions. The projection of the MD structures into the sub-space defined by the largest principal components recapitulates the major differences between structures, highlighting possible relationships between different conformational states.

For the case described in this paper, the PCA calculation was carried out on the C-alphas of the various experimental structures, using only those regions for which homologous residues are found in all structures, based on sequence alignment.

### Energy decomposition method

The Energy Decomposition Method is based on the calculation of the interaction matrix *M*_*ij*_, which is determined by evaluating average, interresidue, non-bonded (van der Waals and electrostatics) interaction energies between residue pairs, calculated over all structures visited during an MD trajectory. For a protein of *N* residues, this calculation yields an *N*×*N* matrix of pair couplings *m*_*ij*_ such that the total average non-bonded energy of the protein is given by the sum over the matrix entries. We showed that, after diagonalizing the matrix *M*_*ij*_, one can approximate pair couplings using the first eigenvalue lambda and eigenvector w:

(4) $$\begin{array}{l} E_{fold}=\sum_{i,j\;=\;1}^{N}m_{ij}\;=\;\;\sum_{i,j\;=\;1}^{N}\sum_{k\;=\;1}^{N}\lambda_{k}w_{i}^{k}w_{j}^{k}\\ \;\approx \;\sum_{i,j\;=\;1}^{N}\lambda_{1}w_{i}^{1}w_{j}^{1} \end{array}$$

The first eigenvector profile reports on the single residue contributions to the essential stabilization energy.

In this context, it must be underlined that the spirit of the method is to provide a simplification of the noisy energy matrix obtained from MD simulations (the noisy character being due to the intrinsic complexity of the energy interactions, given by the superposition of many comparable atomic pair interactions). The *N* components of the eigenvector associated with the lowest eigenvalue have been shown to identify residues behaving as strong, attractive interaction centers, characterized by components with an intensity higher than the threshold value corresponding to a “flat” normalized vector whose residues would all provide the same contribution. The method thus provides an effective energy approximation, which focuses on the main determinants of stabilization of a certain fold, neglecting highly repulsive interactions (which clearly characterize the eigenvectors associated to the higher eigenvalues). It must be noticed that EDM allows to take into account the modulation of interaction energies due to different structural and dynamical contexts that might not be caught by using more simplified energy models, neglecting motional aspects of protein conformations.

The method was validated against experimental data and a relationship was found between the topological and energetic properties of a protein and its stability (Genoni et al., 2010; Morra et al., 2010a, 2014; Torella et al., 2010).

In the case of multidomain proteins, which cannot be described by one single eigenvector, EDM was extended to take into account the minimal number of essential eigenvectors necessary to describe the stability of each (sub)domain and of the whole protein. This is based on the concept of domain as a compact and independent folding unit. In particular, starting from the analysis of the non-bonded interaction energy matrix associated with whole protein, our method filters out and selects only those specific subsets of interactions that define possible independent folding nuclei within a complex protein structure. This allows grouping different protein fragments into energy clusters that are found to correspond to structural domains (Genoni et al., 2012).

Therefore, in analogy with the case of one-domain proteins, we postulate that: (a) for each domain there should exist only one associated eigenvector recapitulating the most significant interactions of the domain, in the same way as the eigenvector associated with the lowest eigenvalue recapitulates the most significant protein interactions for single-domain proteins; (b) each “domain eigenvector” should have a block structure, namely the significant components of each “domain eigenvector” should correspond to the residues belonging to the domain; (c) the union of all the significant blocks should cover all the protein residues. In an ideal case the previous hypotheses would be satisfied and, after selecting the proper set of eigenvectors, the “filtered” non-bonded interaction energy matrix would be simply obtained as,

(5) $$E^{fold}\;=\sum_{i\;=\;1}^{N_{D}}\lambda_{i}v_{i}v_{i}^{T}$$

with *N*_*D*_ as the number of protein domains, and it would have a block structure that enables to immediately identify the protein subunits. In fact, in an ideal situation of non-interacting (or low-interacting) domains the matrix **E**^*fold*^ (from now on, also referred to as “essential folding matrix”) completely neglects the inter-domain interactions whereas each of its blocks represents the essential folding interactions of the corresponding domain.

In a real situation, the eigenvectors of the non-bonded interaction energy matrix **E**^*nb*^ are characterized by overlapping blocks of significant components and more than one eigenvector for each domain is generally needed. In order to satisfy as much as possible the working hypotheses introduced above, it is necessary to select the smallest set of eigenvectors that cover the largest part of residues (i.e., components) with the minimum redundancy. Hence, the corresponding essential folding matrix is obtained as

$$E^{fold}\;\approx E_{\;approx}^{\;fold}\;=\;\sum_{i\;=\;1}^{N_{e}}\lambda_{i}v_{i}v_{i}^{T}$$

where *N*_*e*_, namely the number of essential eigenvectors, is generally > *N*_*D*_. The matrix **E**^*fold*^ is afterwards further filtered through a symbolization process to emphasize the significant non-bonded interactions and, finally, it is subject to a proper clustering procedure leading to the domains identification.

The goal of the analysis is a matrix whose relevant elements correspond to the essential components of the protein non-bonded interaction energy matrix, components that allow to identify the most important residues in defining and stabilizing the three dimensional organization of the different sequences. In turn, this gives insights into the determinants of the structural stability of the detected domains.

In the case of Pol seven eigenvectors were used.

The full procedure for the selection of essential eigenvectors is described in Genoni et al. J. Phys. Chem. B 2012 (Genoni et al., 2012).

## Author contributions

GC, designed research, ran experiments, wrote the paper; MM, designed research, ran experiments, analyzed results; MS, wrote the paper, analyzed results; TC, analyzed results, wrote the paper; AK designed research, wrote the paper.

### Conflict of interest statement

The authors declare that the research was conducted in the absence of any commercial or financial relationships that could be construed as a potential conflict of interest.

## Supplementary material

Table S1 **PDB names of the Pol structures used to define the principal directions of motion**.

Figure S1 **Representative RMSD matrix calculated by comparing the structures sampled during the open to the ones sampled during the closed simulations**. It is to be noted that the labels on the axes indicate the number of the sampled frame.

Figure S2 **Essential conformational spaces spanned by the simulations starting from the closed or ajar conformations in binary complex with only DNA (in the absence of complementary nucleotide)**.

Figure S3 **MM-PBSA energies calculated on the ensembles of structures of the most representative cluster from each simulation**.

Figure S4 **Representations of the main interaction and flexibility vectors (from the closed and open simulations) used to define the principal networks of interaction in the protein**.

Figure S5 **Characterization of the essential subspaces covered by the various simulations after reweighting**.
