# Supplementary material for: DNA Polymerase Conformational Dynamics and the Role of Fidelity-Conferring Residues: Insights from Computational Simulations
Source: Front Mol Biosci. 2016 May 27;3:20. doi: 10.3389/fmolb.2016.00020 (PMC4882331; doi:10.3389/fmolb.2016.00020)
Supplement: Table S1 — PDB names of the Pol structures used to define the principal directions of motion. [file Table1.DOCX]

**Supplementary Material for**

DNA polymerase conformational dynamics and the role of fidelity-conferring residues: Insights from computational simulations

Massimiliano Meli,^1^ Marko Sustarsic,^2^ Timothy D. Craggs,^2^ Achillefs N. Kapanidis,^2,^* Giorgio Colombo^1,^*

1) Istituto di Chimica del Riconoscimento Molecolare, CNR. Via Mario Bianco 9, 20131 Milano, Italy

2) Biological Physics Research Group, Department of Physics, Clarendon Laboratory, University of Oxford, Parks Road, Oxford OX1 3PU, UK

Corresponding authors: [kapanidis@physics.ox.ac.uk](mailto:kapanidis@physics.ox.ac.uk); [g.colombo@icrm.cnr.it](mailto:g.colombo@icrm.cnr.it)

**Table S1.** PDB names of the Pol structures used to define the principal directions of motion.

|  | PDB Code | Position in the 2D  Principal Subspace (Fig 2) |
| --- | --- | --- |
| 1 | 1LV5 | -X -Y |
| 2 | 2HHW | -X -Y |
| 3 | 2HVH | -X -Y |
| 4 | 3EZ5 | -X -Y |
| 5 | 3HP6 | -X -Y |
| 6 | 3HT3 | -X -Y |
| 7 | 3PV8 | -X -Y |
| 8 | 3PX0 | -X -Y |
| 9 | 3PX4 | -X -Y |
| 10 | 3PX6 | -X -Y |
| 11 | 3THV | -X -Y |
| 12 | 3TI0 | -X -Y |
| 13 | 4DQI | -X -Y |
| 14 | 4DQQ | -X -Y |
| 15 | 4DS4 | -X -Y |
| 16 | 4DS5 | -X -Y |
| 17 | 4DSE | -X -Y |
| 18 | 4DSF | -X -Y |
| 19 | 4DSJ | -X -Y |
| 20 | 4EZ9 | -X -Y |
| 21 | 4F2R | -X -Y |
| 22 | 4F2S | -X -Y |
| 23 | 4F3O | -X -Y |
| 24 | 4F4K | -X -Y |
| 25 | 4F8R | -X -Y |
| 26 | 2HVI | -X +Y |
| 27 | 3HPO | -X +Y |
| 28 | 4DQP | -X +Y |
| 29 | 4DQR | -X +Y |
| 30 | 1NJW | +X -Y |
| 31 | 1NJX | +X -Y |
| 32 | 1NK0 | +X -Y |
| 33 | 1NK4 | +X -Y |
| 34 | 1NK6 | +X -Y |
| 35 | 1NK8 | +X -Y |
| 36 | 1NKB | +X -Y |
| 37 | 1U47 | +X -Y |
| 38 | 1UA1 | +X -Y |
| 39 | 1XC9 | +X -Y |
| 40 | 4B9M | +X -Y |
| 41 | 4B9N | +X -Y |
| 42 | 4B9S | +X -Y |
| 43 | 4B9T | +X -Y |
| 44 | 4B9U | +X -Y |
| 45 | 4B9V | +X -Y |
| 46 | 4E0D | +X -Y |
| 47 | 1L3S | +X +Y |
| 48 | 1L3T | +X +Y |
| 49 | 1L3U | +X +Y |
| 50 | 1L3V | +X +Y |
| 51 | 1L5U | +X +Y |
| 52 | 1NJY | +X +Y |
| 53 | 1NJZ | +X +Y |
| 54 | 1NK5 | +X +Y |
| 55 | 1NK7 | +X +Y |
| 56 | 1NK9 | +X +Y |
| 57 | 1NKC | +X +Y |
| 58 | 1NKE | +X +Y |
| 59 | 1U45 | +X +Y |
| 60 | 1U48 | +X +Y |
| 61 | 1U49 | +X +Y |
| 62 | 1U4B | +X +Y |
| 63 | 1UA0 | +X +Y |
| 64 | 2BDP | +X +Y |
| 65 | 2HHQ | +X +Y |
| 66 | 2HHS | +X +Y |
| 67 | 2HHT | +X +Y |
| 68 | 2HHU | +X +Y |
| 69 | 2HHV | +X +Y |
| 70 | 2HHX | +X +Y |
| 71 | 2HW3 | +X +Y |
| 72 | 2XO7 | +X +Y |
| 73 | 2XY5 | +X +Y |
| 74 | 2XY6 | +X +Y |
| 75 | 2XY7 | +X +Y |
| 76 | 2Y1I | +X +Y |
| 77 | 2Y1J | +X +Y |
| 78 | 3BDP | +X +Y |
| 79 | 3EYZ | +X +Y |
| 80 | 3TAN | +X +Y |
| 81 | 3TAP | +X +Y |
| 82 | 3TAQ | +X +Y |
| 83 | 3TAR | +X +Y |
| 84 | 4B9L | +X +Y |
| 85 | 4BDP | +X +Y |
| 86 | 4DQS | +X +Y |
| 87 | 4DSI | +X +Y |
| 88 | 4DSK | +X +Y |
| 89 | 4DSL | +X +Y |
| 90 | 4DWI | +X +Y |
| 91 | 4UQG | +X +Y |
