# Supplementary figures and images for: DNA Polymerase Conformational Dynamics and the Role of Fidelity-Conferring Residues: Insights from Computational Simulations
Source: Front Mol Biosci. 2016 May 27;3:20. doi: 10.3389/fmolb.2016.00020 (PMC4882331; doi:10.3389/fmolb.2016.00020)

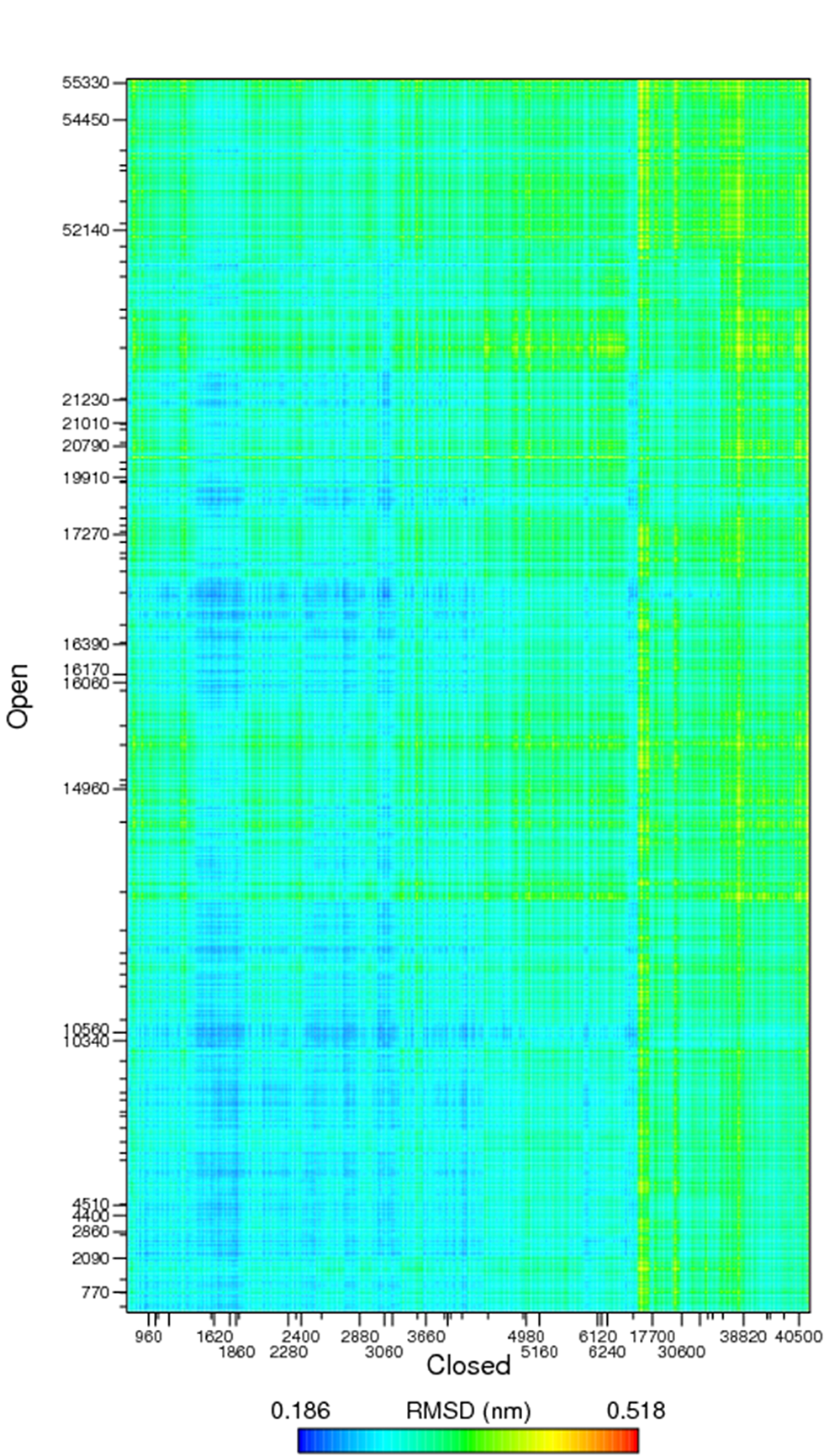

Supplement: Figure S1 — Representative RMSD matrix calculated by comparing the structures sampled during the open to the ones sampled during the closed simulations. It is to be noted that the labels on the axes indicate the number of the sampled frame. [file Image1.TIFF]

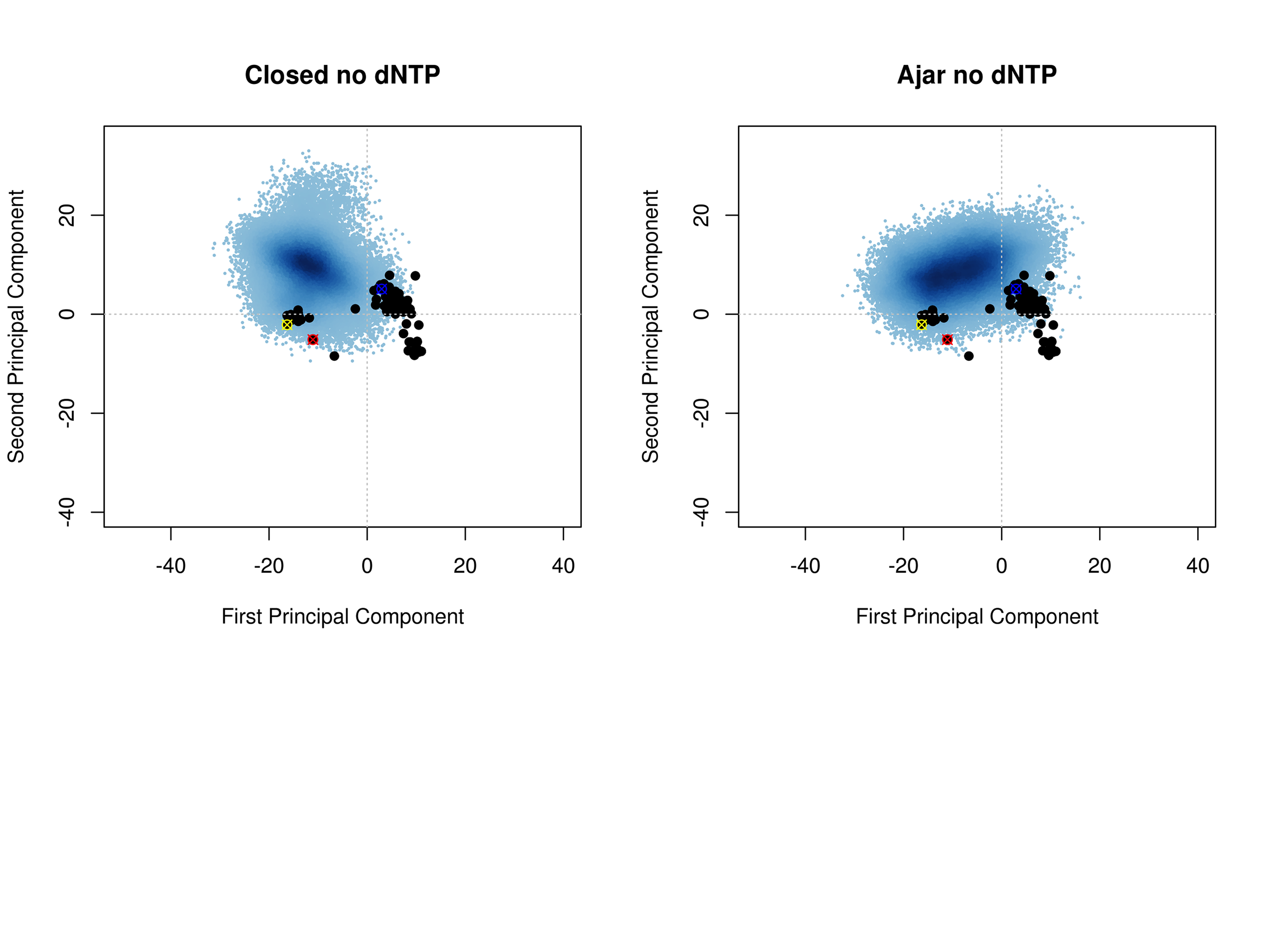

Supplement: Figure S2 — Essential conformational spaces spanned by the simulations starting from the closed or ajar conformations in binary complex with only DNA (in the absence of complementary nucleotide). [file Image2.TIF]

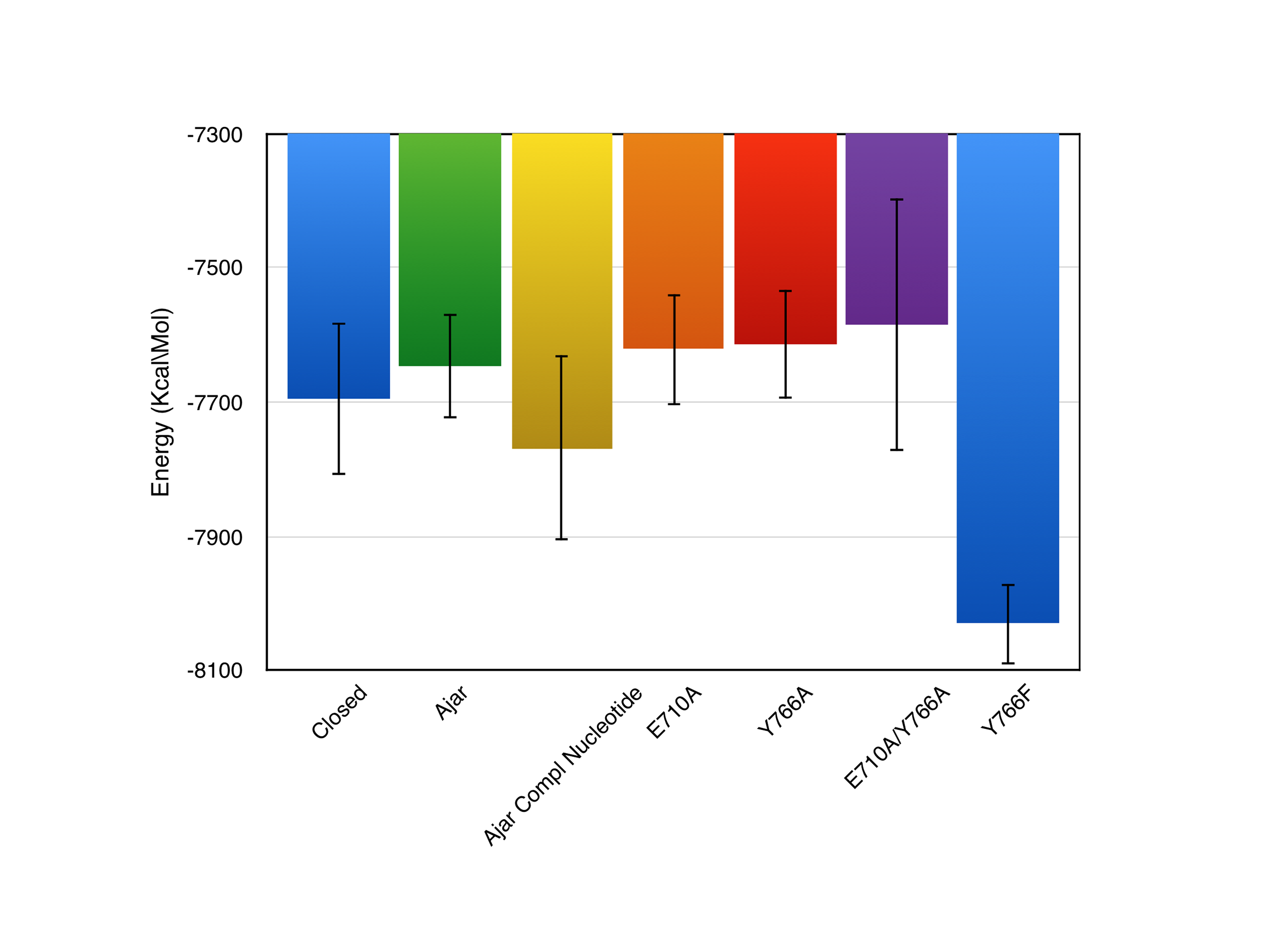

Supplement: Figure S3 — MM-PBSA energies calculated on the ensembles of structures of the most representative cluster from each simulation. [file Image3.TIF]

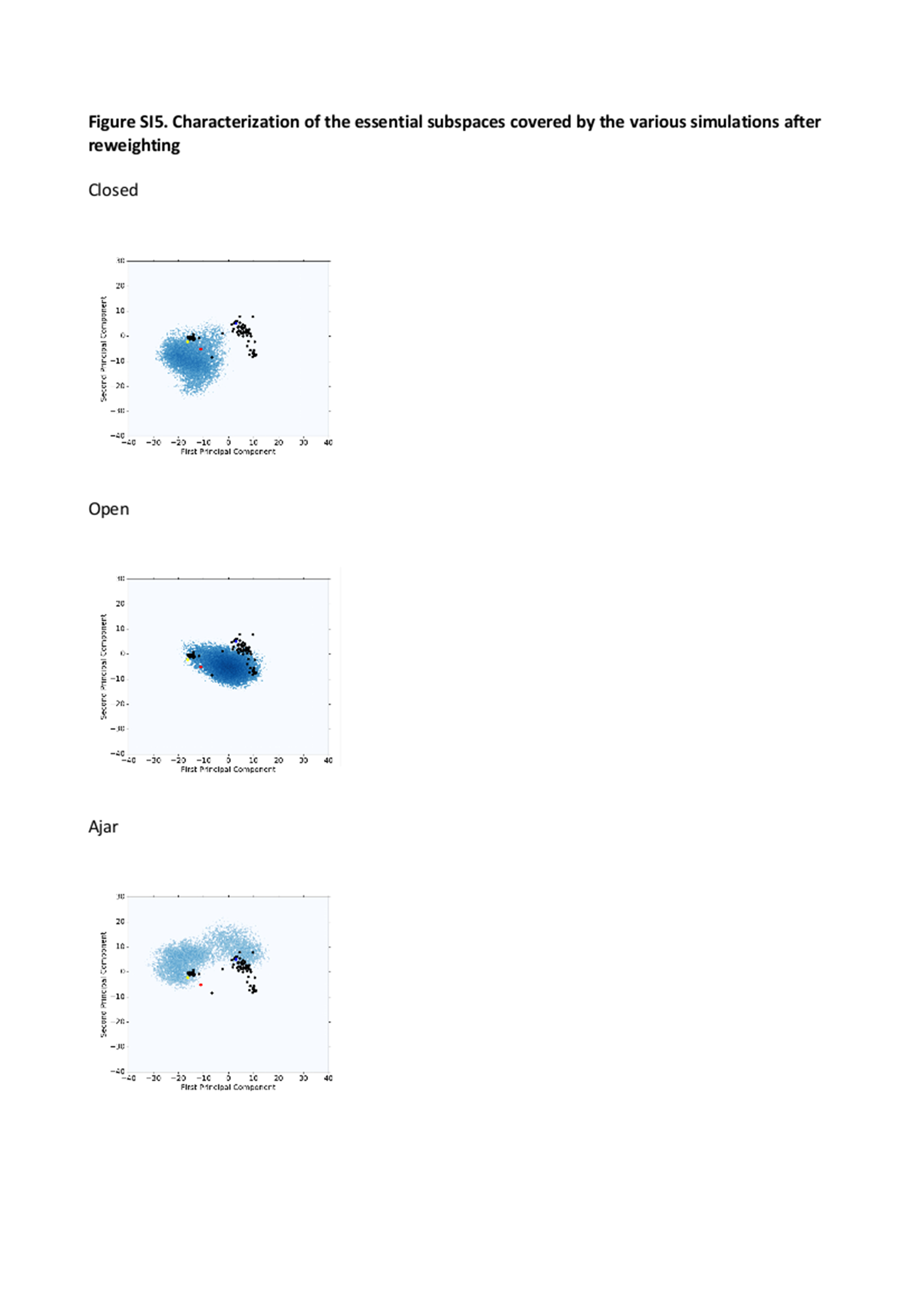

Supplement: Figure S5 — Characterization of the essential subspaces covered by the various simulations after reweighting. [file Image5.tif]
