# Supplementary material for: DNA Polymerase Conformational Dynamics and the Role of Fidelity-Conferring Residues: Insights from Computational Simulations
Source: Front Mol Biosci. 2016 May 27;3:20. doi: 10.3389/fmolb.2016.00020 (PMC4882331; doi:10.3389/fmolb.2016.00020)

Figure SI4. Representations of the main interaction and flexibility vectors (from the closed and open simulations) used to define the principal networks of interaction in the protein.

Closed structure vector sum

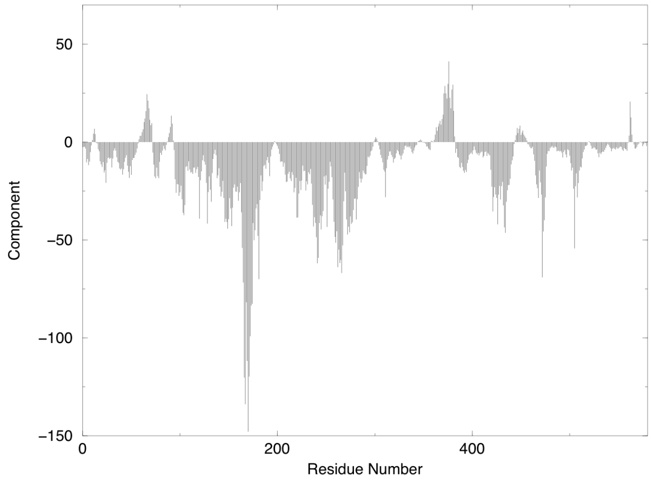

Open structure vector sum

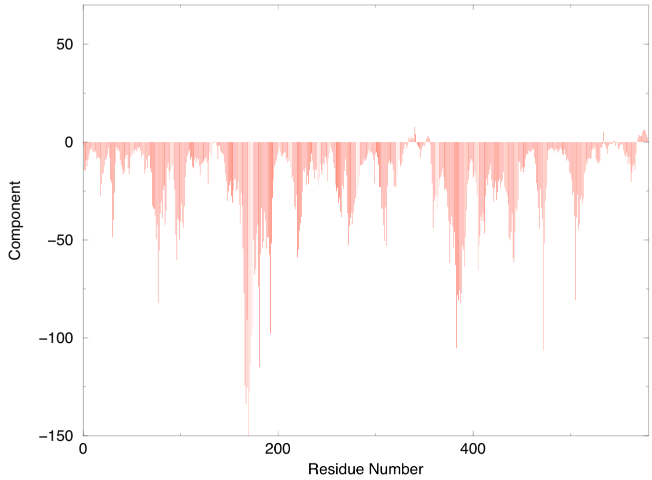

Differential local flexibility analysis (Black, closed; Red, open)

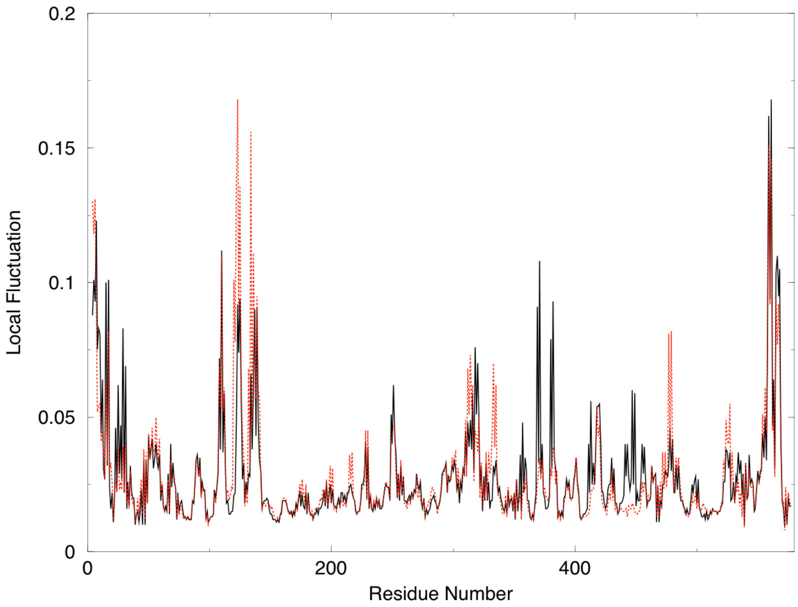

Supplement: Figure S4 — Representations of the main interaction and flexibility vectors (from the closed and open simulations) used to define the principal networks of interaction in the protein. [file Image4.PDF]
